# DEPRESSIVE SYMPTOMS, SLEEP, AND QUALITY OF LIFE IN ADULTS WITH CHRONIC CONDITIONS

**DOI:** 10.1093/geroni/igae098.3284

**Published:** 2024-12-31

**Authors:** Idiatou Diallo, Judy Bass, Rashelle Musci, Leslie Adams, Joseph Gallo, Adam Spira

**Affiliations:** Johns Hopkins University, Baltimore, Maryland, United States; Johns Hopkins University, Baltimore, Maryland, United States; Johns Hopkins University, Baltimore, Maryland, United States; Johns Hopkins University, Baltimore, Maryland, United States; Johns Hopkins Bloomberg School of Public Health, Baltimore, Maryland, United States; Johns Hopkins University, Baltimore, Maryland, United States

## Abstract

Physical, mental, and sleep problems threaten well-being; however, there is limited research on the temporal connections of these issues. We investigated the longitudinal associations among depressive symptoms, sleep disturbances, and quality of life (QoL) among middle-aged and older adults (aged 50+) with diabetes, hypertension, and/or cardiovascular disease in the United States. Participants (N=17,283) drawn from the Health and Retirement Study (HRS) reported at least one of these medical conditions, and data from the 12th (T1), 13th (T2), and 14th (T3) waves (2014-2018) was used. Depressive symptoms were assessed via a modified version of the 8-item Center for Epidemiological Studies-Depression (CES-D) scale (scores: 0-7). Sleep disturbance was measured using the 4-item Jenkins Sleep Scale (JSS) (scores: 2-6). QoL was evaluated through the 5-item Satisfaction with Life Scale (SWLS) (scores: 5-35). Within-person associations showed short-term reciprocal links: higher depressive symptoms predicted higher sleep disturbance (T2–T3: β=0.036, p< 0.05), and higher sleep disturbance predicted higher depressive symptoms (T2–T3: β=0.035, p< 0.01). Higher QoL predicted lower sleep disturbance (T2–T3: β=-0.076, p< 0.001), and higher sleep disturbance predicted lower QoL (T2–T3: β=-0.064, p< 0.001). Within-person associations also indicated a one-way association over time: higher depressive symptoms predicted lower QoL (T1–T2: β=-0.104, p< 0.001; T2–T3: β=-0.141, p< 0.001). Future studies should evaluate the dynamic nature of the associations over longer study periods while assessing the influence of mediators/moderators at different time points. Policies should prioritize integrating sleep, depression, and QoL programs/services into primary care due to their temporal associations among individuals with physical illnesses.

